# Maternal exposure to multiple mycotoxins and adverse pregnancy outcomes: a prospective cohort study in rural Bangladesh

**DOI:** 10.1007/s00204-023-03491-7

**Published:** 2023-04-17

**Authors:** Nicholas N. A. Kyei, Jillian L. Waid, Nurshad Ali, Benedikt Cramer, Hans-Ulrich Humpf, Sabine Gabrysch

**Affiliations:** 1 Institute of Public Health, Charité – Universitätsmedizin Berlin, corporate member of Freie Universität Berlin and Humboldt-Unversität zu Berlin, Charitéplatz 1, 10117 Berlin, Germany; 2 Heidelberg Institute of Global Health, Heidelberg University, Im Neuenheimer Feld 324, 69120 Heidelberg, Germany; 3 Research Department 2, Potsdam Institute for Climate Impact Research (PIK), Member of the Leibniz Association, P. O. Box 60 12 03, 14412 Potsdam, Germany; 4 Helen Keller International–Bangladesh Country Office, House 10E, Road 82, Gulshan 2, Dhaka, 1212 Bangladesh; 5 Department of Biochemistry and Molecular Biology, Shahjalal University of Science and Technology, Sylhet, Bangladesh; 6 Institute of Food Chemistry, Westfälische Wilhelms-Universität Münster, Corrensstr. 45, 48149 Münster, Germany

**Keywords:** Mycotoxins, Human biomonitoring, Exposure assessment, Pregnant women, Birth outcomes, Food safety

## Abstract

There is limited and inconsistent evidence, primarily from cross-sectional studies, linking mycotoxins to adverse birth outcomes. This study investigates the potential role of maternal dietary exposure to multiple mycotoxins in the development of several adverse pregnancy and birth outcomes. We analyzed data from 436 singleton pregnancies enrolled in a prospective cohort study in the rural Habiganj district, Bangladesh, between July 2018 and November 2019. Thirty-five urinary mycotoxin biomarkers were quantified using liquid chromatography coupled with tandem mass spectrometry and used to estimate dietary mycotoxin exposure. Multivariable regression models, adjusted for potential confounding and clustering, were fitted to assess the associations between maternal exposure to frequently occurring mycotoxins (ochratoxin A-OTA, citrinin- CIT, and Deoxynivalenol- DON) and pregnancy loss, preterm birth (PTB), low birth weight (LBW), born small-for-gestational-age (SGA) and small-vulnerable newborn. The results indicate that only in 16 of 436 pregnancies (4%) were urine samples free from all investigated mycotoxins. Biomarkers for six major mycotoxins were detected in the urine samples. OTA (95%), CIT (61%), and DON (6%) were most frequently detected, with at least two mycotoxins co-occurring in the majority of women (63%). There was evidence that maternal dietary intake of OTA was associated with higher odds of having an LBW baby, with the odds increasing in a dose-dependent manner. We found no evidence of associations between pregnancy loss, PTB, SGA, small-vulnerable newborns, and maternal dietary exposure to OTA, CIT, and DON, albeit with large confidence intervals, so findings are consistent with protective as well as large harmful effects. Exposure to multiple mycotoxins during pregnancy is widespread in this rural community and represents a health risk for mothers and babies. Tailored public health policies and interventions must be implemented to reduce mycotoxin exposure to the lowest possible level.

**Supplementary Information:**

The online version contains supplementary material available at 10.1007/s00204-023-03491-7.

## Introduction

In most developing countries, a substantial proportion of pregnancies end in miscarriage, stillbirth, or live birth with adverse pregnancy outcomes, such as low birth weight (LBW), small for gestational age (SGA), and preterm birth (PTB) (Lee et al. 2013). Newborns with these adverse outcomes have increased risks of morbidity and mortality during the perinatal periods, impaired growth and neurodevelopment, and increased health and development risks throughout their lifetime (Christian et al. 2013; Katz et al. 2013).

While the causes of most adverse pregnancy outcomes are multifactorial, maternal nutrition undoubtedly impacts the pregnancy’s development and the newborn's well-being (Ota et al. 2014). Most considerations for the optimum maternal diet focus on dietary diversity and balancing quantity and quality. However, besides insufficient nutrient content, foods consumed by expectant mothers may contain hazardous contaminants such as mycotoxins. Mycotoxins are natural toxins produced as secondary metabolites of micro-fungi that are harmful to humans and animals even in low concentrations (Bennett and Klich 2003). Mycotoxins contaminate a significant proportion of dietary staples and food crops worldwide (Eskola et al. 2020), especially in countries with warm and humid climatic conditions and poor agricultural and storage practices (Ali 2019; Bennett and Klich 2003; Omotayo et al. 2019). As mycotoxins are also resistant to several processing and cooking practices (Bullerman and Bianchini 2007; Raters and Matissek 2008), pregnant women in low-income settings who consume monotonous diets based on frequently contaminated staple foods are at increased risk of chronic mycotoxin exposures, with potentially serious health consequences (Marin et al. 2013; Turner et al. 2012).

Several studies from low- and middle-income countries (LMICs) report high occurrences and concentrations of major mycotoxins such as aflatoxins (AFs), fumonisin B_1_ (FB1), ochratoxin A (OTA), citrinin (CIT), zearalenone (ZEN), and deoxynivalenol (DON) in pregnant women (Chan-Hon-Tong et al. 2013; Groopman et al. 2014; Kyei et al. 2020; Piekkola et al. 2012). With evidence of transplacental transfer of mycotoxins such as AFs, OTA, CIT, DON, and ZEN to the developing fetus in humans and animals (Goyarts et al. 2007; Nielsen et al. 2011; Partanen et al. 2010; Reddy et al. 1982; Warth et al. 2019; Woo et al. 2012), maternal exposure to these mycotoxins during pregnancy represents a potentially harmful exposure during the critical developmental phase of a child’s life (Groopman et al. 2014; Ismail et al. 2021).

Most observational studies so far have provided inconsistent evidence for causal relationships between single mycotoxin exposures (frequently aflatoxin B_1_) and higher occurrence of adverse birth outcomes in LMICs (Andrews-Trevino et al. 2021; Kyei et al. 2020). With the wide variation in detected mycotoxin concentrations, sample sizes, and possible confounding factors adjusted for in these studies, it is not surprising that some found associations and others did not. For example, some studies indicate that exposure to higher AF levels was associated with an increased risk of adverse birth outcomes such as LBW and SGA, while others provide no evidence or mixed evidence for such relationships (Andrews-Trevino et al. 2019; Kyei et al. 2020; Passarelli et al. 2020). Given that many mycotoxins require similar climatic and environmental conditions for production, maternal dietary exposure to concurrent multiple mycotoxins can be expected (Turner et al. 2012; Vidal et al. 2018), and this may result in synergistic toxic effects (Smith et al. 2016). However, most epidemiological studies investigating linkages between mycotoxins and human health have focused on the independent effects of single mycotoxin exposure without considering the potential presence of other mycotoxins.

Consequently, there is a need for further research to improve our understanding of the combined or adjusted effects of multiple mycotoxin exposure on adverse birth outcomes. However, longitudinal studies evaluating such effects are scarce. To the best of our knowledge, only a recent study in rural Ethiopia investigated the effect of maternal exposure to concurrent mycotoxins during pregnancy on adverse pregnancy outcomes (Tesfamariam et al. 2022). In Bangladesh, previous biomonitoring studies analyzing urine samples from pregnant women in Bangladesh report the frequent occurrence of AFM_1_, OTA, CIT, and DON (Ali et al. 2015, 2017, 2016). Nevertheless, associations between multiple mycotoxin exposures during pregnancy and adverse birth outcomes have yet to be documented. Using data from a prospective cohort study in the rural Habiganj district, we aimed to quantify maternal mycotoxin exposures during pregnancy, estimate the burden of specific adverse pregnancy outcomes and investigate the potential role of exposure to multiple mycotoxins for the development of these adverse pregnancy outcomes in rural Bangladesh.

## Materials and methods

The study followed the Strengthening the Reporting of Observational Studies in Epidemiology-nutritional epidemiology (STROBE-nut) checklist (Lachat et al. 2016).

### Study setting, participants, and study design

This study was embedded in the *Food and Agricultural Approaches to Reducing Malnutrition* (FAARM) study, a cluster-randomized controlled trial conducted in two sub-districts of Habiganj district under Sylhet division in Bangladesh (ClinicalTrials.gov ID: NCT02505711). FAARM included 2705 young married women in 96 settlements (geographic clusters) who reported an age below 30 years, an interest in gardening, and had access to at least 40 m^2^ of land. Settlements were randomized into 48 intervention and 48 control clusters. The FAARM trial evaluates the impact of a homestead food production program implemented by the international non-governmental organization Helen Keller International on undernutrition in young children. Further information on the FAARM trial design is available in the study protocol (Wendt et al. 2019).

**Fig. 1:**
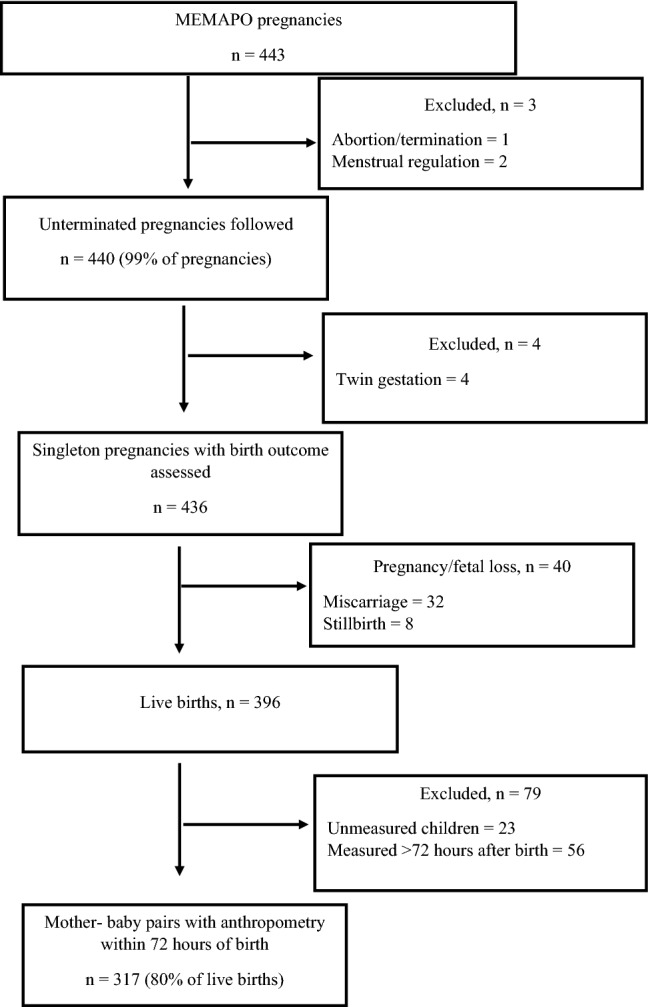
Flow chart of the MEMAPO cohort study

As an add-on to the FAARM trial, we conducted a prospective cohort study: *Maternal Exposure to Mycotoxins and Adverse Pregnancy Outcomes* (MEMAPO). MEMAPO enrolled a subsample of FAARM women during early pregnancy. FAARM women were given calendars to record the first day of each menstrual period. Those with a known last menstrual period (LMP) that suggested a gestational age of < 20 weeks were considered eligible for MEMAPO. A total of 443 pregnancy events were identified through FAARM’s active house-to-house bi-monthly surveillance between July 2018 and November 2019. Pregnant women were consented to by field workers in the local language, and those consenting gave a urine sample for mycotoxin analysis. The MEMAPO open cohort followed up with pregnant women until the end of pregnancy to investigate the role of maternal exposure to mycotoxins for the development of specific adverse pregnancy outcomes (miscarriage, preterm birth, low birth weight, and birth small-for-gestational age). Trained field data collectors attempted to visit all newborn babies within 72 h of delivery for anthropometric measurements (weight, length, head circumference) using standardized equipment (infant scale, infantometer board, and insertion tape). For the current study, we analyzed data from 436 non-terminated singleton pregnancy events and 317 babies measured within 72 h of delivery (Fig. 1).

### Background characteristics of the rural pregnant cohort

Data on descriptive characteristics, such as a woman’s religion, height, highest education level, and household wealth, were extracted from the FAARM baseline and endline surveys conducted in 2015 and 2019, respectively. Using the FAARM endline survey’s asset module, the household’s relative position within the 2014 Demographic and Health Survey (DHS) national wealth quintiles was calculated using the Equity Tool (Metrics for Management 2016; NIPORT et al. 2016). Data on other descriptive characteristics, such as maternal weight and household food security status, were extracted from the FAARM routine surveillance round when the woman was enrolled in MEMAPO. The food security status of the pregnant woman’s household was assessed using the Household Food Insecurity Access Scale (HFIAS) developed by the Food and Nutrition Technical Assistance Project (FANTA) and others (Coates et al. 2007). The HFIAS questionnaire assesses household economic access to food, food preferences, anxiety about household food supply, and food quantity within a 30-day recall period and is used to distinguish between food-secure and food-insecure households (Coates et al. 2007; Jones et al. 2013).

Information on women’s current and previous pregnancies, including gravidity, parity, miscarriages, stillbirths, antenatal attendance, and iron supplementation, was extracted from FAARM endline and surveillance datasets. The woman’s age at pregnancy enrollment was calculated from her age at the FAARM baseline survey. The season of enrollment was categorized as pre-monsoon hot (March–May), rainy monsoon (June–October), and cool, dry winter (November–February) according to the sampling date. Women’s average dietary diversity scores were calculated using data from all surveillance rounds. Dietary diversity is the variety of foods pregnant women consume from ten food groups. These food groups include starchy staples, vitamin A-rich vegetables and fruits; dark green leafy vegetables; other vegetables; other fruits; meat, poultry, and fish; eggs and pulses/legumes; nuts and seeds; and dairy products (FAO and FHI 360 2016). A woman who consumes foods from five or more groups of the 10 food groups is considered to have adequate dietary diversity.

### Maternal dietary mycotoxin exposure assessment during pregnancy

A detailed description of the methods used for quantifying maternal dietary mycotoxin exposure and risk assessment for this pregnant cohort has been published elsewhere (Kyei et al. 2022). Briefly, potential maternal multiple mycotoxin exposure was assessed from first-morning urine samples collected during the first half of pregnancy (up to 20 weeks gestation). Urine samples were screened for 35 mycotoxin biomarkers and quantified by a validated “dilute and shoot” method using high-performance liquid chromatography-tandem mass spectrometry detection, according to Gerding et al. (2014). Instrumental limits of all analytes in urine (Supplementary Table S1) were determined in a range of 0.01 to 30 ng/ml for the limit of detection (LOD) and 0.03 to 100 ng/ml for the limit of quantification (LOQ). Left-censored urine mycotoxin data, i.e. concentrations below the LOD and LOQ of the analytical method, were imputed using the substitution methods as recommended in the European Food Safety Authority (EFSA) guidelines (EFSA 2010). For this study, the middle-bound substitution scenario was used; results below LOD and LOQ were substituted with the value of LOD/2, and LOQ/2, respectively. Dietary exposure assessments were performed by estimating probable daily intake (PDI) from urine mycotoxin concentrations for the frequently occurring mycotoxins (OTA, CIT, and DON), corrected for urine density using the formula (Solfrizzo et al. 2014):

$$PDI (\frac{ng}{kg}bw)=\left[C*\frac{V}{W*E}\right]*100,$$

where *C* is the mycotoxin concentration, adjusted for urine density or creatinine, W is the individual’s body weight (kg), *E* is the excretion rate (%) of the mycotoxin, and *V* is the daily urine excretion volume (ml)(Kyei et al. 2022). PDI estimates for the middle-bound exposure scenario were used to investigate the relationship between dietary mycotoxin intake and birth outcomes. For DON and CIT, where both parent compounds and metabolites were detected, exposure assessment calculations were performed on total DON (DON + DON-15GlcA + DON-3GlcA) and total CIT (CIT + Dihydrocitrinone (HO-CIT)) by converting metabolites using their molar mass.

Details of health risk characterization of the estimated maternal daily intake of individual mycotoxins based on their toxicity profiles are presented elsewhere (Kyei et al. 2022). Briefly, considering OTA’s carcinogenicity, no exposure level is considered safe. Thus, the Margin of Exposure (MOE) approach was used for health risk assessment. MOE was computed as a ratio of the benchmark dose lower confidence limit (BMDL_10_) for OTA and estimated maternal PDI of OTA. The scientific committee of EFSA and the WHO consider a MOE of 10,000 or more to be of low concern for public health (EFSA 2013). BMDL_10_ value for neoplastic effects OTA of 14.5 µg/kg bw/day (EFSA CONTAM Panel (EFSA Panel on Contaminants in the Food Chain) et al. 2020) was used for MOE estimations. For CIT and DON, the estimated PDIs were compared with established health-based guidance values to calculate a hazard quotient (HQ), i.e. the proportion of the population who consume these mycotoxins above established tolerable daily intake (TDI) levels. The TDI values for CIT of 200 ng/kg bw/day and for DON of 1000 ng/kg bw/day were those derived by scientific committees, such as the Joint FAO/WHO Expert Committee on Food Additives (JECFA) and the EFSA (EFSA 2012, 2017; FAO/WHO 2011). When the HQ was less than one, the exposure was considered to be within safe limits.

### Birth anthropometry and adverse birth outcomes

Information on the date of birth, the reason for the end of pregnancy, newborn sex, birth weight, length, and head circumference were obtained from the FAARM birth surveillance system. Early ultrasound assessments of fetal gestational age were not feasible in this study. Gestational age (GA) at delivery was calculated based on self-reported last menstrual period (LMP) and the child's date of birth. Furthermore, GA estimates based on head circumference measurements within 72 h of delivery were calculated using an excel-based gestational age calculator (IG21 2022) developed by the International Fetal and Newborn Growth Consortium for the 21st Century (INTERGROWTH-21st) and compared to the LMP-based estimates. Where the difference in the two estimated gestational ages was more than ± 14 days, the LMP-based assessment was deemed incorrect and replaced with the biometric-based estimate. Based on the INTERGROWTH-21st international newborn size standards (Villar et al. 2014), we created gestational age-adjusted birth weight z-scores (weight-for-age z-scores).

Our primary dependent variables included pregnancy loss and three established indicators of newborn vulnerability—preterm birth (PTB), low birth weight (LBW), and birth small-for-gestational-age (SGA). These three indicators (PTB, LBW, and SGA) were assessed individually and combined to describe five identified vulnerable newborn phenotypes (Ashorn et al. 2020). Babies born at least at term with a birth weight of about 2.5 kg or more and not considered SGA were classified as not vulnerable (Ashorn et al. 2020). Standard recommended definitions for adverse birth outcomes were used (WHO 1977). LBW was defined as birth weight < 2.5 kg, regardless of gestational age or sex. SGA was defined as birth weight < 10th percentile, adjusted for gestational age and sex based on the INTERGROWTH-21st standards. PTB was defined as being born alive before 37 weeks of gestation (259 days). Miscarriage and stillbirths were considered together as pregnancy loss.

### Covariates

Covariates included in our multivariable models were chosen a priori based on relevant literature (Islam Pollob et al. 2022; Khan et al. 2018; Kiserud et al. 2017; Shah et al. 2014; Tamirat et al. 2021; Zhou et al. 2016). Our models included the following potential confounders: maternal age (years), height (cm), weight (kg), education level (six ordered categories), parity (*n*), history of pregnancy loss (yes; no), antenatal attendance (yes; no), iron supplementation (yes; no), infant sex (male, female), household wealth index (quintiles, with first two groups collapsed), average dietary diversity score (*10 food group scale*), and food security status (food secure; food insecure). For LBW and SGA, the following binarized variables: low maternal weight (maternal weight < 55 kg) and short maternal stature (maternal height < 145 cm) were used as predictors, respectively, based on literature (Black et al. 2013; Mumbare et al. 2012). Furthermore, we controlled our analyses for the presence of other detected mycotoxins and clustering at the settlement and woman levels. We also controlled for each season as a fixed effect covariate (pre-monsoon hot; rainy monsoon; cool-dry winter).

### Statistical analysis

Descriptive statistics are presented as mean ± SD or median [interquartile range (IQR)] for quantitative variables and as frequencies and percentages for categorical variables. Mixed-effect linear regression models were used to determine if maternal mycotoxin exposure was associated with GA at delivery, birth weight, birth length, and head circumference while accounting for other factors. Mixed effect binary logistic regression models were used to identify if maternal mycotoxin concentrations were associated with pregnancy loss, PTB, SGA, and small-vulnerable newborn status while accounting for other factors. Models were fit using mycotoxin exposure as a continuous variable after a zero-skewness logarithmic transformation (Stata command *lnskew0*) (Stata Corporation 2021) or a categorical variable using tertiles. Tertiles were defined based on the distribution of mycotoxin exposure levels, and the lowest tertile (T1) was assigned as the reference. Additionally, the highest tertile of OTA was broken into finer groups to describe further the relationship between adverse pregnancy outcomes and higher OTA intake. To assess a linear trend, we additionally treated the tertile values as a continuous variable in the regression model.

In addition, the PDI of OTA, CIT, and DON were categorized into dichotomous variables to depict high exposure compared with established health-based guidance values by scientific committees, such as the Joint FAO/WHO Expert Committee on Food Additives (JECFA) and the EFSA (EFSA 2012, 2017; FAO/WHO 2011). Estimated consumption above the tolerable daily intake (TDI) values for CIT of 200 ng/kg bw/day was classified as high exposure. Only 4 women had estimated consumption of DON above the TDI for DON of 1000 ng/kg bw/day. Thus, a high intake of DON was defined as an intake of more than half the TDI of DON. There is currently no health-based guidance value for OTA due to its carcinogenicity (EFSA 2020), and all the exposed women had a Margin of Exposure (MOE) of health concern (i.e. benchmark dose lower confidence limit (BMDL_10_) for OTA / estimated PDI of OTA < 10,000). Thus, to define relatively high exposure of OTA, we explored the best functional form that described the relationship between PDI of OTA and LBW. A 1000 ng/kg bw/day cut-off was determined as the exponential curve’s turning point that best fit the data.

To avoid over-adjustment and multi-collinearity across the covariates, different measures of collinearity were assessed using Stata’s collin command. Variance inflation factor < 6, tolerance < 0.01, and condition number > 10 were considered acceptable. Data management and statistical analysis were performed using Stata version 17.0 (StataCorp LLC, College Station, TX, USA).

## Results

A study flow chart is presented in Fig. 1. We obtained 443 urine samples from 439 pregnant women (8 women with two pregnancies during the study period) who consented to enroll in the MEMAPO cohort and had expected delivery dates before the FAARM birth surveillance ended. Of the 443 pregnancy events, three women (0.7%) who had their pregnancies terminated or underwent menstrual regulation were excluded from further analyses. We also excluded 4 women (0.9%) with twin gestation from further analyses. We followed up on the remaining 436 singleton pregnancy events until the end of pregnancy. These pregnancies resulted in 396 live births, of which 317 (80%) were measured within 72 h of delivery.

### Maternal characteristics and pregnancy outcomes

The baseline characteristics of the 436 pregnant women with unterminated singleton pregnancies are shown in Table 1. Three-quarters were Muslim, and one-quarter were Hindu. Five years after the reference period, almost 80% came from households belonging to the middle and upper wealth quintiles of the 2014 Bangladesh Demographic and Health Survey wealth index (NIPORT et al. 2016), while a tenth belonged to the wealthiest quintile and another tenth to the lower two wealth quintiles. On average, pregnant women were 27 ± 4 years old at enrollment, and their median parity was 2 before the current pregnancy event (IQR: 1, 4). Only 6% of women had completed secondary school or higher degrees, while 14% had no formal education. More than two-fifths (44%) of women were from food-insecure households, and the majority (62%) had suboptimal dietary diversity (i.e. less than five varieties of food from ten food groups). The women weighed 47 kg on average at the time of enrollment, at around 15 weeks of gestation (IQR: 29, 78). The gestational age at the end of pregnancy was 37.6 ± 7 weeks. Of the 436 pregnancies, 396 (91%) resulted in a live birth, 32 (7%) in a miscarriage, and 8 (2%) in a stillbirth.

**Table 1:** Description of maternal characteristics, pregnancy outcomes, and average dietary mycotoxin intake for the cohort of pregnant women in rural Habiganj district, Bangladesh (*N* = 436)

Characteristic	*n*	Percent
**Religion**		
Muslim	327	75%
Hindu	109	25%
**Household wealth quintile (as in DHS 2014)**^1^		
Poorest	6	1%
Lower	41	9%
Middle	170	39%
Upper	175	40%
Wealthiest	44	10%
**Household food security status**		
Food secure	242	56%
Mildly/moderately food insecure	110	25%
Severely food insecure	194	19%
**Highest education level**		
No formal education	63	14%
Partial primary	119	27%
Complete primary	98	22%
Partial secondary	133	31%
Completed secondary	12	3%
Post-secondary	11	3%
**Adequate dietary diversity (≥ 5 of 10 food groups)**		
Yes	165	38%
**Gravidity before enrollment**		
Nulligravida	9	2%
Primigravida	80	18%
Multigravida	347	80%
**Parity at enrollment**		
Nulliparous	13	3%
Primiparous	110	25%
Multiparous	313	72%
**Previous stillbirth/miscarriage**		
Yes	155	36%
**Any antenatal attendance**		
Yes	252	58%
**Iron supplementation**		
Yes	181	42%
**Season of enrollment**		
Mar-May: pre-monsoon hot	101	23%
Jun-Oct: rainy monsoon	244	56%
Nov-Feb: cool, dry winter	91	21%
**Outcome of pregnancy**		
Miscarriage	32	7%
Stillbirth	8	2%
Live birth	396	91%
**Mycotoxin intake of health concern**		
Ochratoxin A	436	100%
Citrinin	74	17%
Deoxynivalenol	4	1%
**Continuous variables**	Mean (SD)	Range
Woman’s age at enrollment (years)	27 (4)	18–38
Woman’s height (cm)	150 (6)	135–170
Woman’s weight at enrollment (kg)	47 (8)	29–78
Gestational age at the end of pregnancy (weeks)	37.6 (7)	8–48
Average dietary diversity score, ten food groups (MDDW)	4.6 (1)	2–8
PDI of OTA, ng/kg body weight	410 (462)	11–3968
PDI of CIT, ng/kg body weight	132 (268)	4–2885
PDI of DON, ng/kg body weight	231(293)	48–4967

^1^This is not a relative wealth quintile, but the estimate of the households’ national wealth quintile if the assets owned in 2019 were in the 2014 DHS survey (constructed using www.equitytool.org)

*SD* Standard deviation, *MDDW* Minimum dietary diversity for women, *PDI* Provisional daily intake, *OTA* Ochratoxin A, *CIT* Citrinin, *DON* Deoxynivalenol

### Child characteristics and birth outcomes

The characteristics of the 317 children measured within 72 h of delivery are shown in Table 2. There were slightly more male children than females (54% vs. 46%). At birth, the children weighed 2.7 ± 0.4 kg, had a head circumference of 34 ± 1 cm, and measured 47 ± 2 cm in length. Overall, 17% of the newborns (95% CI 13–22) were born preterm, 26% were LBW (95% CI 31–41), 36% were SGA (95% CI 31–41), and half of them were born with a small-vulnerable newborn phenotype (95% CI 44–56).

**Table 2:** Description of child characteristics and adverse birth outcomes for the cohort of pregnant women in rural Habiganj district, Bangladesh (*N* = 317)

Child characteristic	*n*	Proportion	95% confidence interval
**Sex**			
Male	170	54%	48–59
Female	147	46%	41–52
**WHO classification of gestational age at birth**			
Preterm: less than 259 days (37 weeks)	54	17%	13–22
Term: 259–293 days (37–41 weeks)	255	80%	76–84
Post-term: 294 days (42 weeks) or more	8	3%	1–5
**Low birth weight**			
Yes	81	26%	20–31
**Small-for-gestational age**			
Yes	113	36%	31–41
**Vulnerable newborn phenotypes**			
AGA-T-NBW (not vulnerable)	158	50%	44–56
AGA-PT-NBW	17	5%	3–9
AGA-PT-LBW	29	9%	6–13
SGA-T-NBW	63	20%	16–24
SGA-T-LBW	42	13%	10–18
SGA-PT-LBW	8	3%	1–5
**Small-vulnerable newborn**			
Yes	159	50%	44–56
**Continuous variables**	Mean	SD	Range
Birth weight (kg)	2.7	0.4	1.3–4.0
Birth length (cm)	47	1.6	38.6–51.8
Head circumference (cm)	34	1.2	27.7–37.6

*AGA* Appropriate-for-gestational-age, *SGA* Small-for-gestational age, *T* Term birth, *PT* Preterm birth, *NBW* Normal birthweight (≥ 2500 g), *LBW* Low birth weight (< 2500 g)

### Maternal dietary mycotoxin exposure

**Fig. 2:**
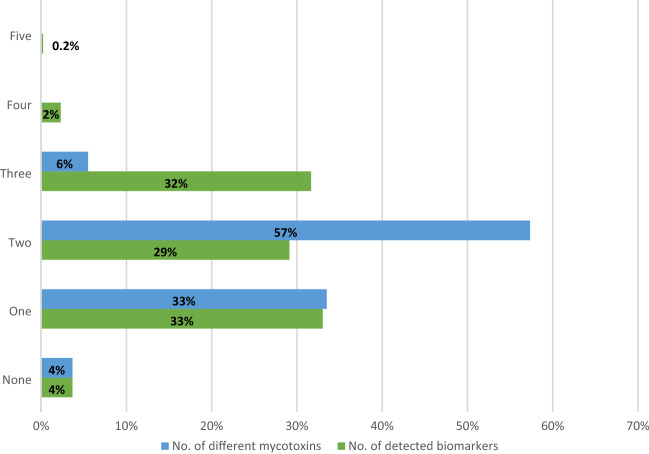
Number of detected biomarkers and mycotoxins among 436 pregnant women in rural Bangladesh. Only 16 pregnant women (4%) had no detectable biomarkers of investigated mycotoxins. A third of the women had one detectable mycotoxin biomarker, about a third had two detectable biomarkers, and another third had three detectable mycotoxin biomarkers. Only 2% contained more than three. This corresponds to two different mycotoxins detected in over half and three in 5% of the women, while a third had one detectable mycotoxin

**Fig. 3:**
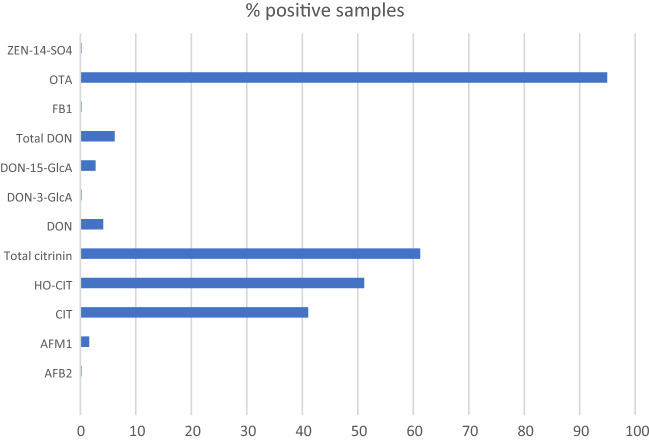
Occurrence of mycotoxin biomarkers in urine samples of 436 pregnant women in rural Habiganj district, Bangladesh. OTA was the most frequently occurring mycotoxin detected in 95% of the maternal urine samples. Total CIT was detectable in 61% of the urine samples, while total DON was detectable in 6%. *AFM1* Aflatoxin M1, *AFB2* Aflatoxin B2, *CIT* Citrinin, *DON* Deoxynivalenol, *DON*-*15*-*GlcA* Deoxynivalenol-15-glucuronide, *DON-3-GlcA* Deoxynivalenol-3-glucuronide, *FB1* Fumonisin B1, *HO*-*CIT* Dihydrocitrinone, *ZEN-14-SO4* Zearalenone-14-sulfate. Positive samples refer to samples containing the analyte above the limit of detection; total CIT (CIT + HOCIT); total DON (DON + DON-Glucuronides)

**Fig. 4:**
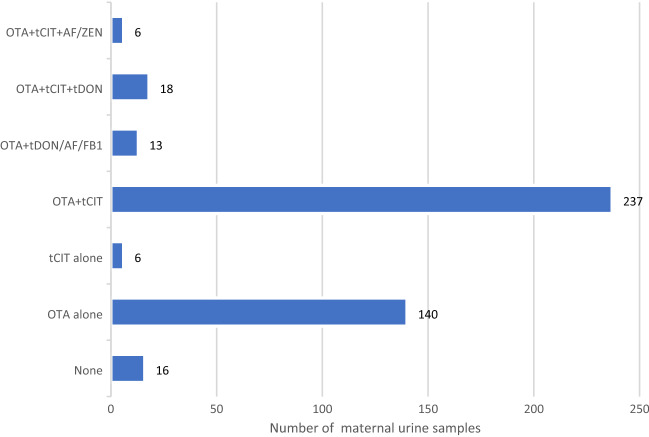
Mycotoxin co-occurrence among 436 pregnant women in rural Bangladesh. Biomarkers for six major mycotoxins (AF, CIT, DON, FB1, OTA, and ZEN) were detected in the maternal urine samples. Co-occurrence of mycotoxins was detected in 274/436 (63%) pregnant women. Co-occurrence of OTA and CIT was the prevailing co-exposure (54%). *AF* Aflatoxins M1, *CIT* Citrinin, *DON* Deoxynivalenol, *FB*_*1*_ Fumonisin B_1_, *HO*-*CIT* Dihydrocitrinone, *ZEN* Zearalenone, *tCIT* total CIT (CIT + HO-CIT), *tDON* total DON (DON + DON-Glucuronides), *AF* (AFM1 + AFB2)

Overall, ten different biomarkers were detected out of the 35 urinary mycotoxin biomarkers investigated. The detected biomarkers represented six major mycotoxins: AF, CIT, DON, FB_1_, OTA, and ZEN. Only 4% of the pregnant women included in this study had no detectable levels of the investigated mycotoxin biomarkers. About a third of each were exposed to one, two, and three detectable mycotoxin biomarkers (Fig. 2). OTA was the most frequently occurring mycotoxin detected in 95% of maternal urine samples. Total citrinin was detectable in 61% of urine samples, while total DON was detectable in 6% (Fig. 3). Co-occurrence of OTA and CIT was the prevailing mycotoxin exposure, present amongst 54% of pregnant women (Fig. 4). Under a moderate exposure scenario, maternal dietary exposure to OTA, CIT, and DON was of public health concern in 100%, 17%, and 1% of the pregnant women, respectively. The estimated mean dietary exposure to the three frequently detected mycotoxins among the pregnant cohort in this study was for OTA 410 ng/kg bw ± 462, for CIT 132 ng/kg bw ± 268, and for DON 231 ng/kg bw ± 293 (Table 1). Overall, estimated daily intake of OTA, CIT, and DON was classified as “high” in 8% (35/436), 17% (74/436), and 5% (20/436) of the pregnant women, respectively (Tables 3 and 4).

**Table 3:** Association between anthropometric birth outcomes and maternal exposure to frequently occurring mycotoxins in rural Habiganj district, Bangladesh (*N* = 317)

Mycotoxin intake	GA at delivery (weeks)	Birth weight (g)	Birth length (cm)	Head circumference (cm)
	Adjusted^*a*^β (95% CI)	Adjusted^*b*^ β (95% CI)	Adjusted^*c*^ β (95% CI)	Adjusted^*c*^ β (95% CI)
**PDI of OTA**				
Ln (PDI)	0.077 (− 0.162, 0.316)	− 16 (− 44, 21)	0.080 (− 0.071, 0.232)	− 0.001 (− 0.059, 0.057)
Tertiles (ng/kg bw)				
T1 (11–174)	Reference	Reference	Reference	Reference
T2 (175–400)	0.309 (− 0.227, 0.845)	25 (− 48, 97)	− 0.083 (− 0.429, 0.262)	− 0.022 (− 0.154, 0.110)
T3 (401–3968)	0.186 (− 0.370, 0.741)	− 10 (− 85, 65)	0.159 (− 0.194, 0.511)	− 0.016 (− 0.151, 0.118)
*P* for trend	0.553	0.752	0.329	0.826
High PDI of OTA (> 1000 ng/kg/day)				
No	Reference	Reference	Reference	Reference
Yes (*n* = 26)	− 0.192 (− 0.943, 0.560)	− 101 (− 203, − 0.1)	0.289 (− 0.194, 0.772)	0.076 (− 0.108, 0.260)
**PDI of CIT**				
Ln (PDI)	− 0.065 (− 0.206, 0.075)	11 (− 10,32)	0.014 (− 0.074, 0.103)	0.011 (− 0.025, 0.048)
Tertiles (ng/kg bw)				
T1 (4–21)	Reference	Reference	Reference	Reference
T2 (22–75)	** − **0.008 (− 0.533, 0.518)	− 28 (− 101, 46)	0.123 (− 0.204, 0.450)	− 0.049 (− 0.174, 0.076)
T3(75–2885)	− 0.081 (− 0.566, 0.404)	10 (− 72, 93)	− 0.060 (− 0.368, 0.247)	− 0.060 (− 0.177, 0.058)
*P* for trend	0.738	0.800	0.684	0.322
High PDI of CIT (> 1000 ng/kg/day)				
No	Reference	Reference	Reference	Reference
Yes (*n* = 60)	− 0.019 (− 0.588, 0.550)	60 (− 16, 136)	− 0.149 (− 0.511, 0.214)	0.078 (− 0.060, 0.216)
**PDI of DON**				
Ln (PDI)	0.035 (− 0.157, − 0.227)	7 (− 28, 42)	0.041 (− 0.078, 0.161)	− 0.157 (− 0.061, 0.030)
Tertiles (ng/kg/day)				
T1 (48–140)	Reference	Reference	Reference	Reference
T2(141–214)	0.204 (− 0.305, 0.713)	− 10 (− 80, 61)	0.162 (− 0.158, 0.482)	− 0.039 (− 0.162, 0.083)
T3(215–4967)	− 0.110 (− 0.572, 0.352)	− 23 (− 97, 50)	0.085 (− 0.197, 0.368)	− 0.023 (− 0.130, 0.085)
*P* for trend	0.582	0.529	0.586	0.703
High PDI of DON (> 500 ng/kg/day)				
No	Reference	Reference	Reference	Reference
Yes (*n* = 14)	0.089 (− 0.507, 0.685)	62 (− 61, 185)	0.283 (− 0.107, 0.674)	− 0.120 (− 0.269, 0.028)

*GA* Gestational age, *Ln* Zero-skewness natural logarithm, *PDI* Provisional daily intake in ng/kg bw/day, *OTA* Ochratoxin A, *CIT* Citrinin, *DON* Deoxynivalenol

^*a*^Adjusted for mother’s age at enrollment, household wealth index, mother’s educational attainment, mother’s weight at enrollment, average dietary diversity score, household food security status, parity, history of previous pregnancy loss, season of sample collection, infant sex, detection of more than one mycotoxin and clustering at the settlement and woman levels

^*b*^Adjusted for all covariates above^*a*^, plus length at birth and gestational age at birth

^*c*^Adjusted for all covariates above^*b*^, plus mother’s height

**Table 4:** Association between adverse birth outcomes and higher maternal dietary intake of ochratoxin A, citrinin, or deoxynivalenol among the cohort of pregnant women in rural Habiganj district, Bangladesh

Mycotoxin intake	Pregnancy loss (*N* = 436)	Preterm birth (*N* = 317)	Low birth weight (*N* = 317)	Small for gestational age (*N* = 317)	Small-vulnerable newborn (*N* = 317)
	Adjusted^*a*^ OR (95% CI)	Adjusted^*a*^ OR (95% CI)	Adjusted^*b*^ OR (95% CI)	Adjusted^*c*^ OR (95% CI)	Adjusted^*c*^ OR (95% CI)
**PDI of OTA**					
Ln (PDI)	0.88 (0.58, 1.35)	0.76 (0.52, 1.11)	1.65 (1.06, 2.55)	1.10 (0.80, 1.51)	1.02 (0.76, 1.38)
Tertiles (ng/kg bw)					
T1 (11–174)	Reference	Reference	Reference	Reference	Reference
T2 (175–400)	1.53 (0.61, 3.81)	0.55 (0.25, 1.24)	1.07 (0.40, 2.91)	0.62 (0.31, 1.26)	0.50 (0.26, 1.0)
T3 (401–3968)	0.93 (0.34, 2.50)	0.65 (0.29, 1.44)	2.64 (0.98, 7.10)	1.30 (0.64, 2.66)	1.08 (0.54, 2.19)
*P* for trend	0.920	0.331	0.041	0.372	0.684
High PDI of OTA (> 1000 ng/kg/day)					
No	Reference	Reference	Reference	Reference	Reference
Yes (*n* = 26)	0.24 (0.03, 1.95)	1.10 (0.37, 3.23)	4.01 (1.25, 12.81)	2.25 (0.57, 8.87)	1.86 (0.73, 4.70)
**PDI of CIT**					
Ln (PDI)	0.88 (0.65, 1.18)	1.01 (0.79, 1.29)	0.97 (0.72, 1.30)	1.01 (0.82, 1.24)	0.88 (0.72, 1.07)
Tertiles (ng/kg bw)					
T1 (4–21)	Reference	Reference	Reference	Reference	Reference
T2 (22–75)	1.78 (0.69, 4.60)	0.88 (0.38, 2.06)	2.35 (0.90, 6.12)	1.34 (0.64, 2.80)	1.01 (0.51, 2.03)
T3(75–2885)	0.97 (0.32, 2.93)	0.92 (0.36, 2.32)	1.40 (0.44, 4.49)	1.15 (0.49, 2.66)	0.76 (0.34, 1.66)
*P* for trend	0.988	0.870	0.559	0.784	0.478
High PDI of CIT (> 1000 ng/kg/day)					
No	Reference	Reference	Reference	Reference	Reference
Yes (*n* = 60)	0.46 (0.15, 1.48)	1.17 (0.52, 2.60)	0.45 (0.16, 1.31)	1.08 (0.52, 2.26)	0.91 (0.45, 1.87)
**PDI of DON**					
Ln (PDI)	1.36 (0.84, 2.20)	1.00 (0.65, 1.53)	1.07 (0.66, 1.72)	1.02 (0.71, 1.47)	0.94 (0.67, 1.32)
Tertiles (ng/kg/day)					
T1 (48–140)	Reference	Reference	Reference	Reference	Reference
T2(141–214)	0.98 (0.36, 2.62)	0.81 (0.36, 1.82)	1.21 (0.47, 3.07)	1.30 (0.66, 2.56)	1.17 (0.62, 2.21)
T3(215–4967)	1.40 (0.54, 3.65)	1.22 (0.55, 2.72)	1.17 (0.48, 2.88)	1.23 (0.59, 2.54)	0.96 (0.49, 1.90)
*P* for trend	0.452	0.600	0.739	0.591	0.937
High PDI of DON (> 500 ng/kg/day)					
No	Reference	Reference	Reference	Reference	Reference
Yes (*n* = 14)	2.46 (0.55, 10.93)	0.44 (0.05, 3.70)	4.33 (0.89, 21.10)	0.85 (0.22, 3.32)	0.79 (0.22, 2.78)

*Ln* Zero-skewness natural logarithm, *PDI* Provisional daily intake in ng/kg bw/day, *OTA* Ochratoxin A, *CIT* Citrinin, *DON* Deoxynivalenol

^a^Adjusted for mother’s age at enrollment, household wealth index, mother’s educational attainment, mother’s weight at enrollment, average dietary diversity score, household food security status, parity, history of previous pregnancy loss, the season of sample collection, antenatal attendance, iron supplementation, detection of more than one mycotoxin, and clustering at the settlement and woman levels

^b^Adjusted for all covariates above^a^, low maternal weight (mother’s weight at enrollment < 55 kg), plus infant sex, length at birth, and gestational age at birth

^c^Adjusted for all covariates above^a^, short maternal stature (mother’s height < 145 cm), plus infant sex, length at birth, and gestational age at birth

### Relationships between maternal dietary mycotoxin exposures and adverse birth outcomes

Regression coefficients (β) along with a 95% confidence interval (95% CI) for the associations between anthropometric birth outcomes (GA at delivery, birth weight, birth length, and head circumference) and higher maternal dietary intake of OTA, CIT, and DON are presented in Table 3, controlling for other covariates and adjusting for clustering. The results show, for instance, that newborns with maternal OTA intake in the highest tertile weighed on average 10 g less at birth compared to those with maternal OTA intake in the lowest tertile, while a true difference in birth weight ranging between − 85 g and + 65 g is reasonably likely. Nonetheless, maternal intake of high levels of OTA (> 1000 ng/kg/day) resulted in a 101 g (95% CI 0.1–203; *P* = 0.05) reduction of birth weight compared to newborns whose mothers consumed lower levels of OTA. We did not find evidence for the effect of increasing maternal OTA intake on the duration of pregnancy, birth length, and head circumference. Likewise, using the continuous (log-PDI), categorical (tertiles), and binarized (high vs. low) exposure variables, we found no evidence for an association between higher maternal daily intakes of CIT and DON, and duration of pregnancy, birth weight, birth length, and head circumference at birth.

The odds ratios (ORs) and 95% CIs for the associations between higher maternal dietary intake of OTA, CIT, or DON and adverse birth outcomes, –including pregnancy loss, babies born too soon (PTB), and those born too small (LBW, SGA)– are presented in Table 4, adjusted for detecting two or more mycotoxins, clustering, and other potential confounding factors. When treating the continuous log-PDI of maternal OTA as a linear effect, we found that for each unit increase, the odds of having a low birth weight baby increased by 65% (adjusted OR: 1.65; 95% CI 1.06, 2.55; *P* = 0.026). Using the binary variable, a high maternal dietary intake of OTA (> 1000 ng/kg/day) was associated with a fourfold increase in the odds of having an LBW baby, with an OR between 1.25 and 12.5 reasonably likely (*P* = 0.019). For each tertile increase in maternal OTA intake from lowest to highest, the odds of having an LBW baby increase 1.7-fold (adjusted OR: 1.68; 95% CI 1.02, 2.76; *P* for trend = 0.04). Breaking down the highest tertile into finer categories, the dose-dependent increase in the odds of having an LBW baby becomes much clearer (Supplementary Table S3), increasing from 2.03 (OTA intake: 401–1000 ng/kg/day) to 4.05 (OTA intake: 1001–1500 ng/kg/day) to 6.59 (OTA intake > 1500 ng/kg/day) (*P for trend* = *0.009*). We found no evidence of an association between higher maternal dietary intake of OTA and other adverse birth outcomes, albeit with large uncertainties. For instance, among women with a high intake of OTA, the odds of SGA were more than double, with a halving of the odds and a nine-fold increase also reasonably likely. Similarly, our results provide no evidence of an increasing dose effect of maternal dietary intake of CIT and DON on adverse birth outcomes.

In multivariable logistic regression models investigating fixed-dose effects (i.e. detection above LOD), we found no evidence that maternal exposure to OTA alone versus in combination with other mycotoxins differed in their impact on adverse birth outcomes (Table 5). However, uncertainties were large. For instance, among women with both detectable urinary OTA and CIT, the odds of LBW were 84% higher than among women with detectable OTA alone, while a reduction of 19% and a four-fold increase were also reasonably likely. A more detailed breakdown of adverse birth outcomes by maternal single or co-exposures to specific mycotoxins resulted in categories with too few women or events for statistical inference. Therefore, we present the results descriptively (Supplementary Table S4).

**Table 5:** Fixed-dose effects of maternal single or co-exposures to specific mycotoxins and the development of adverse birth outcomes in the cohort of pregnant women in rural Habiganj district, Bangladesh

Mycotoxins detected above LOD	Pregnancy loss (*N* = 436)	*n* (yes)	Preterm birth (*N* = 317)	*n* (yes)	Low birth weight (*N* = 317)	*n* (yes)	Small for gestational age (*N* = 317)	*n* (yes)	Small, vulnerable baby (*N* = 317)	*n* (yes)
	Adjusted^*a*^ OR (95% CI)		Adjusted^*a*^ OR (95% CI)		Adjusted^*b*^ OR (95% CI)		Adjusted^*c*^ OR (95% CI)		Adjusted^*c*^ OR (95% CI)	
OTA alone	Reference	140 (12)	Reference	100 (15)	Reference	100 (20)	Reference	100 (38)	Reference	100 (50)
OTA + CIT	0.81 (0.35, 1.86)	237 (19)	1.37 (0.69, 2.72)	177 (35)	1.84 (0.81, 4.19)	177 (54)	0.59 (0.32, 1.08)	177 (62)	0.84 (0.47, 1.51)	177 (93)
None and * other combinations	1.60 (0.56, 4.52)	59 (9)	0.66 (0.20, 2.20)	40 (4)	1.32 (0.37, 4.68)	10 (7)	0.57 (0.22, 1.43)	40 (13)	0.64 (0.27, 1.51)	40 (16)

*LOD* Limit of detection, *OTA* Ochratoxin A, *CIT* Citrinin, *other combinations- includes CIT alone, OTA or CIT in combination with deoxynivalenol, aflatoxin, and zearalenone

^a^Adjusted for mother’s age at enrollment, household wealth index, mother's educational attainment, mother’s weight at enrollment, average dietary diversity score, household food security status, parity, history of previous pregnancy loss, the season of sample collection, antenatal attendance, iron supplementation, detection of more than one mycotoxin, and clustering at the settlement and woman levels

^b^Adjusted for all covariates above^a^, low maternal weight (mother’s weight at enrollment < 55 kg), plus infant sex, length at birth, and gestational age at birth

^*c*^Adjusted for all covariates above^a^, short maternal stature (mother’s height < 145 cm), plus infant sex, length at birth, and gestational age at birth

## Discussion

Like in other resource-poor settings, exposure to multiple mycotoxins during pregnancy is common in Bangladesh (Kyei et al. 2022). The present study builds upon a recent human biomonitoring and dietary risk assessment study among a pregnant cohort in rural Bangladesh (Kyei et al. 2022). The aim was to quantitatively describe the relationship between maternal dietary exposures to frequently occurring mycotoxins and the development of adverse birth outcomes. To the best of our knowledge, this is the first epidemiological study to investigate the association of prenatal exposure to multiple mycotoxins and adverse birth outcomes in Bangladesh. Our findings provide evidence for a moderate-to-strong positive association between low birth weight and increasing maternal dietary intake of OTA during pregnancy, having adjusted for potential confounders and the presence of other mycotoxins. We found no evidence for an association between pregnancy loss, PTB, SGA, and small-vulnerable newborns and maternal dietary exposures to OTA, CIT, and DON.

The burden of most adverse birth outcomes observed in our rural cohort in Sylhet division, Bangladesh, was comparable to national and regional estimates. Of the 436 detected singleton pregnancies, 32 (7.3%) ended in a spontaneous miscarriage. This finding is similar to incidence estimates of miscarriage among pregnant women in two prospective Chinese studies (i.e. 7.9 and 7.4%, respectively) (Wang et al. 2003; Zhou et al. 2016). It is, however, noteworthy that the actual number of miscarriages is likely higher because many miscarriages occur very early in pregnancy before the woman might even know she is pregnant. The stillbirth risk of about 2% found in this rural cohort is consistent with the national stillbirth risk of 20 to 25 per 1000 births (Blencowe et al. 2016; Halim et al. 2018). Despite being recognized as the country with the fastest reduction in stillbirth risk in South Asia between 2000 and 2015 (Lawn et al. 2016), Bangladesh still ranks among the top ten countries globally in terms of absolute numbers of stillbirths (Blencowe et al. 2016).

We found that 17% of babies were born preterm in our rural pregnant cohort from Habiganj district in Bangladesh’s Sylhet division. This is only slightly lower than the PTB risks of 19.4% (Baqui et al. 2013) and 22.3% (Shah et al. 2014) found in two previous studies in different rural areas in Sylhet division and the estimated national prevalence of 19.1% (Blencowe et al. 2012).

The prevalence of low birth weight in our rural pregnant cohort (26%) was much higher than the current national estimate of LBW, which is 16% (NIPORT and ICF International 2019). The National Low Birth Weight Survey (NLBWS) of Bangladesh from 2003–2004 (Bangladesh Bureau of Statistics 2005) estimated the prevalence of LBW in Bangladesh at 36%, ranging from 28% in Chittagong Division, over 38% in Sylhet to 44% in Dhaka Division, with substantially higher estimates in rural than in urban areas. Our finding of more than a third of live births (36%) being SGA is somewhat consistent with the South Asian regional estimate of about 45% of live births in 2010 (Lee et al. 2013).

Although there are national, regional, and global estimates for the prevalence of PTB (Blencowe et al. 2012), LBW (Blencowe et al. 2019), and SGA (Lee et al. 2017), which are established indicators of newborn vulnerability, there are no estimates that assess these conditions together to describe different small-vulnerable newborn phenotypes (Ashorn et al. 2020). Using these more detailed phenotypes has been shown to help in identifying babies at the highest risk of mortality (Paixao et al. 2021). The current study is thus the first to describe the different small-vulnerable newborn phenotypes and estimate their individual and combined burden in rural Bangladesh. These estimates will serve as a first point of reference that can help public health authorities set priorities, develop and implement appropriate interventions, and measure changes from their interventions.

The finding that only 16 of 436 women with singleton pregnancies (4%) were free from all investigated mycotoxin biomarkers indicates that pregnant women have widespread exposure to mycotoxins in this rural community in Bangladesh. Considering that different mold species may contaminate a given food sample and that molds often require similar conditions for toxin production (Frisvad et al. 2006), it is not unexpected that concurrent exposure to multiple mycotoxins was predominant in this community. Due to methodological challenges, no prospective human studies so far have investigated the cumulative effect of co-exposure to multiple mycotoxins or the independent effect of exposure to specific mycotoxins while controlling for the presence of other mycotoxins. The only human study investigating the link between maternal exposure to multiple mycotoxins and two adverse birth outcomes (PTB and SGA) in rural Ethiopia (Tesfamariam et al. 2022) did not provide any statistical evidence for associations. Besides the limited number of adverse birth outcomes, the study defined exposure to a mycotoxin as a serum biomarker concentration above or equal to the detection limit. Thus, like most previous studies on single mycotoxins and pregnancy outcomes (Kyei et al. 2020), a dose–response relationship was not assessed. Furthermore, the high occurrence of concurrent exposure to multiple mycotoxins in the Ethiopian study (Tesfamariam et al. 2022) reduced the power to detect any true effects due to the low number of unexposed women, similar to our study. All pregnant women in the Ethiopian cohort were co-exposed to at least five mycotoxin biomarkers. Fumonisins (detected in > 93% of samples) and tenuazonic acid (detected in 81% of samples) were the most frequently occurring mycotoxins in that setting (Tesfamariam et al. 2022), while OTA was detected in about 32%, CIT in about 37%, and DON in about 39% of maternal urine samples.

Our multivariable regression models, which controlled for the presence of other mycotoxins and a wide range of potential confounders, also showed no evidence of an association between maternal dietary exposures to the most frequently occurring mycotoxin in this rural cohort, OTA (95%), and pregnancy loss, PTB, SGA, and small-vulnerable newborns. However, exposure was generally high. Under a moderate exposure scenario, the mean maternal PDI of OTA was up to 24 times higher than the previous health-based guidance value of 17.1 ng/kg bw (EFSA 2006). Using the recommended margin of exposure (MOE) approach due to the genotoxicity of OTA (EFSA 2020), all the 436 women with singleton pregnancies in this cohort had MOE values below 10,000, indicating a significant concern for the carcinogenic effects of OTA in the study population. Even under the lowest exposure scenario, maternal dietary exposure to OTA, CIT, and DON has been estimated to be of public health concern in 95%, 16%, and 1% of all MEMAPO women, respectively (Kyei et al. 2022).

Animal studies have established that dietary exposure to OTA and CIT causes reduced maternal weight gain, impaired reproduction, reduced fetal weights and crown-to-rump lengths, and teratogenic effects (Malir et al. 2013; Singh et al. 2007). However, the potential consequences of maternal exposure to these toxins in humans remain largely unknown. While a cross-sectional study from Nigeria reported higher OTA levels in babies with LBW compared to normal-weight babies (0.5 vs. 0.9 ng/ml; p = 0.06) and lower mean birth weight for girls exposed to OTA compared to unexposed girls (Jonsyn et al. 1995; Kyei et al. 2020), this is the first prospective study to provide evidence for an increasing dose effect of maternal dietary exposure to OTA on low birth weight.

Concerning DON, our study provides no evidence for such a link: our results were compatible with an effect on LBW, but they were also compatible with the association being due to chance: While we observed 4.3-fold higher odds of LBW for women with an estimated DON intake above 500 ng/kg/day (vs. below), an odds ratio between 0.9 and 21 was also reasonably likely. As few women were exposed to DON in our cohort, the power to detect any true effects of DON exposure on adverse pregnancy outcomes was low. Nonetheless, experimental animal studies have shown that chronic low-dose exposure to DON may cause adverse effects such as anorexia, poor maternal weight gain, immunotoxicity, impaired reproduction, and decreased offspring weight (Pestka 2010; Pestka and Smolinski 2005).

As intestinal epithelial cells are the first targets of ingested mycotoxins such as OTA, CIT, and DON, the mechanism of growth faltering is likely from mycotoxin-induced damage and inflammation of the intestinal mucosa resulting in poor maternal uptake and retention of nutrients leading to poor maternal weight gain and, consequently, growth faltering in the fetus (Ghareeb et al. 2015; Liew and Mohd-Redzwan 2018; Nakayama et al. 2018; Qing et al. 2022; Zhai et al. 2021). Thus, it is somewhat baffling that the evidence for the association between LBW and high OTA intake in our study did not exist for other adverse pregnancy outcomes, such as SGA, which should follow a similar biological pathway. Despite the observed higher odds, the confidence interval was wide and included the null value. Hence, future studies with larger sample sizes or a meta-analysis (Zhao et al. 2022) of similar small studies would provide more precise estimates and improve our understanding of the relationship.

Our multivariable models investigating the fixed-dose effect of single or concurrent exposure to specific mycotoxins were inconclusive. While our findings are compatible with chance effects, they are also compatible with a higher risk for adverse birth outcomes among pregnant women exposed to a combination of mycotoxins as compared to single mycotoxins. Preliminary evidence from in vitro models indicates that while exposure to single mycotoxins at low doses may be non-toxic, exposure to different combinations of toxins at equal amounts may result in complementary or synergistic toxicity (Föllmann et al. 2014; Wan et al. 2013). In our study, the low subgroup sample sizes when considering single and various combinations of mycotoxin exposure seriously hampered the possibility of investigating this further. Nonetheless, the general picture supports the hypothesis that exposure to different combinations of toxins in equal amounts may result in complementary, synergistic, or even antagonistic toxicity. It thus should be further explored in future research.

Notably, the most frequently detected mycotoxins in this population, OTA and CIT, are produced mainly by certain *Aspergillus*, *Monascus*, and *Penicillium* species, which often contaminate food under poor post-harvest handling and storage conditions (Alshannaq and Yu 2017; Frisvad et al. 2006). Several strategies exist for mitigating dietary exposure to these mycotoxins from the food supply in low-income settings (Aldred and Magan 2004; Amuzie et al. 2015; Magan and Aldred 2005, 2007; Matumba et al. 2021). Yet, the rural community lacks adequate knowledge of the conditions that favor mold contamination of crops and the strategies for prevention during crop production and storage (Kyei et al. 2021). This knowledge gap needs to be urgently addressed by implementing appropriate interventions.

This study’s strengths include the relatively large number of pregnant women followed (*n* = 436), along with paired birth surveillance data (*n* = 317 within 72 h of delivery), and the estimation of maternal dietary intake of mycotoxins based on multiple urinary mycotoxin biomarker analyses. The longitudinal design ensures the temporality of exposure is established and thus allows for a causal investigation of the effect of prenatal exposure to multiple mycotoxins (OTA, CIT and DON) on birth outcomes. We also investigated different mycotoxin exposure effects, including fixed-dose effects, and increasing-dose effects, controlling for the presence of other mycotoxins. Furthermore, our adjustment for the design effect and many important potential confounders reduce the potential for bias. Nonetheless, as with all observational studies, residual confounding from unmeasured confounders (e.g. other environmental exposures and maternal morbidity during pregnancy) cannot be ruled out. The applied analytical method for detecting urinary mycotoxins covered a broad spectrum of compounds but focused on the previously detected highly relevant mycotoxins OTA and CIT/HO-CIT (Gerding et al. 2015). Thus, for other analytes, a moderate sensitivity was accepted as more sensitive methods with comparable efficiency were unavailable at the time of sample analysis (Schmidt et al. 2021). Consequently, the LODs of some mycotoxins were comparably high, allowing only the detection of very high exposure scenarios but not the baseline level of mycotoxin uptake in rural Bangladesh (Kyei et al. 2022). Yet, only 4% of pregnant women in our study had no detectable mycotoxins. Hence the relatively high LODs should not have impacted our analysis comparing high with low exposures. Nonetheless, due to the limited sensitivity of the analysis approach, urinary AFM_1_ detection was far lower than described in another pregnant cohort from the Dhaka district (Ali et al. 2017). Consequently, we could not investigate the potential effect of aflatoxin exposure on adverse pregnancy outcomes. Furthermore, while urine sampling is non-invasive and thus convenient for population-based biomonitoring studies, the daily variation in urine composition and excretion rate among individuals adds some difficulties. However, we adjusted for inter-individual urine volume variations by correcting mycotoxin concentrations for urine density and creatinine before dietary exposure estimation.

As in most low-income settings, assessment of gestational age by early ultrasound was not feasible in our rural study area. Generally, gestational age assessment based on a mother's recall of her last menstrual period alone is error-prone and could result in misclassified pregnancies (Savitz et al. 2002). However, the availability of menstrual calendars for women participating in the FAARM trial and the bi-monthly recall period helped to reduce the usual recall errors associated with LMP-based pregnancy dating. In addition, the addition of newborn biometric measurement allowed for an improvement in our gestational dating.

Another limitation of this study is that mycotoxin exposures were only measured at a single point early in pregnancy. Thus, observed exposures may not accurately reflect the mycotoxin exposure throughout pregnancy. Nevertheless, as people often eat monotonous diets of frequently contaminated staple crops, the observed mycotoxin exposures likely provide a fair estimation of usual exposure levels. Notwithstanding, studies collecting bio-samples and fetal biometry at different stages of pregnancy are required to improve our knowledge of specific windows of fetal vulnerability to mycotoxins. Lastly, our findings are based on the cohort of pregnant women from rural households in Habiganj district, Bangladesh, who participated in the FAARM trial, and the conclusions cannot necessarily be generalized to other areas. However, our mycotoxin exposure results and adverse birth outcome prevalence were consistent with those from previous studies in Bangladesh, suggesting that our study population is typical for rural settings in Bangladesh. Thus, such findings may also apply to similar settings. Generally, except for genetic and nutritional differences, which may also vary by location, it is likely that humans can be affected in the same way by specific mycotoxins.

The results of this study add to the limited body of evidence for the potentially harmful effect of early life exposure to mycotoxins. Overall, the following conclusions can be drawn:

- Pregnant women in the MEMAPO prospective cohort study in rural Bangladesh were frequently exposed to multiple mycotoxins, including OTA, CIT, and DON, for which evidence of their health impact on human pregnancy and fetal growth has been lacking or limited.
- Maternal dietary exposure to OTA during pregnancy may lead to increased odds for newborns with LBW in a dose-dependent manner.
- Large uncertainties remain regarding the relationship between maternal dietary exposure to OTA, CIT, or DON and pregnancy loss, PTB, SGA, and small-vulnerable newborns, requiring further investigation.

Although mycotoxins cannot be eliminated entirely from food supplies, appropriate policies should be developed to improve food safety, including implementing bespoke measures to reduce mycotoxin exposure to the minimum, especially among pregnant women.

## Supplementary Information

Below is the link to the electronic supplementary material.

Supplementary file1 (DOCX 36 KB)

## Data Availability

The data presented in this study are available upon reasonable request from the corresponding author.
