# Supplementary material for: Maternal exposure to multiple mycotoxins and adverse pregnancy outcomes: a prospective cohort study in rural Bangladesh
Source: Arch Toxicol. 2023 Apr 17;97(6):1795–812. doi: 10.1007/s00204-023-03491-7 (PMC10182942; doi:10.1007/s00204-023-03491-7)
Supplement: Supplementary file 1 — Supplementary file1 (DOCX 36 KB) [file 204_2023_3491_MOESM1_ESM.docx]

**Supplementary Table S1: Instrumental limits of mycotoxin biomarker concentrations in urine in ng/ml**

| **35 Mycotoxin biomarkers** | **LOD** | **LOQ** |
| --- | --- | --- |
| Aflatoxin B_1_ (AFB_1_) | 0.06 | 0.2 |
| Aflatoxin B_2_ (AFB_2_) | 0.04 | 0.12 |
| Aflatoxin M_1_ (AFM_1_) | 0.1 | 0.3 |
| Aflatoxin G_1_ (AFG_1_) | 0.7 | 2 |
| Aflatoxin G_2_ (AFG_2_) | 0.2 | 0.6 |
| Alternariol (AOH) | 3 | 10 |
| Alternariol monomethyl ether (AME) | 0.3 | 1 |
| Altenuene (ALT) | 1.7 | 5 |
| Beauvericin (BEA) | 1 | 2.5 |
| Citrinin (CIT) | 0.17 | 0.5 |
| Deoxynivalenol (DON) | 1.7 | 5 |
| Deoxynivalenol-3-glucuronide (DON-3-GlcA) | 2.5 | 7.5 |
| Deoxynivalenol-15-glucuronide (DON-15-GlcA) | 2.5 | 7.5 |
| Dihydrocitrinone (HO-CIT) | 0.1 | 0.3 |
| Enniatin B (ENB) | 0.025 | 0.075 |
| Enniatin B_1_ (ENB_1_) | 0.125 | 0.375 |
| Enniatin A (ENA) | 0.06 | 0.175 |
| Enniatin A_1_ (ENA_1_) | 0.12 | 0.35 |
| Fumonisin B_1_ (FB_1_) | 1 | 3 |
| Fumonisin B_2_ (FB_2_) | 30 | 100 |
| HT2-toxin (HT2) | 8 | 25 |
| HT2-toxin-3-glucuronide (HT-2-3-GlcA) | 1.7 | 5 |
| HT2-toxin-4-glucuronide (HT-2-4-GlcA) | 1.3 | 4 |
| Hydroxy-Ochratoxin A (OH-OTA) | 0.05 | 0.15 |
| Ochratoxin A (OTA) | 0.02 | 0.06 |
| 2'R-Ochratoxin A (2'R-OTA) | 0.01 | 0.03 |
| Ochratoxin alpha (OTalpha) | 0.2 | 0.6 |
| T2-toxin (T2) | 0.2 | 0.6 |
| Zearalenone (ZEN) | 0.7 | 2 |
| Zearalenone (ZAN) | 1.25 | 3.75 |
| Zearalanone-14-glucuronide (ZAN-14-GlcA) | 7 | 20 |
| Zearalenone-14-glucuronide (ZEN-14-GlcA) | 7 | 20 |
| alpha-Zearalenol-14-glucuronide (alpha-ZEL-14) | 3 | 10 |
| beta-Zearalenol-14-glucuronide (beta-ZEL-14) | 7 | 20 |
| Zearalenone-14-sulfate (ZEN-14-SO_4_) | 0.3 | 1 |

LOD: Limit of Detection; LOQ: Limit of quantification

**Supplementary Table S2. Quantitative description of the tertiles of estimated probable daily intake of three frequently occurring mycotoxins among a pregnant cohort in rural Bangladesh (N=436)**

| Mycotoxin Tertiles |  | PDI (ng/kg body weight) | | | | | | |
| --- | --- | --- | --- | --- | --- | --- | --- | --- |
|  | n | Mean | SD | Min | Max | p25 | p50 | p75 |
| OTA |  |  |  |  |  |  |  |  |
| Tertile 1 | 146 | 93 | 42 | 11 | 174 | 57 | 92 | 126 |
| Tertile 2 | 145 | 277 | 63 | 175 | 400 | 220 | 278 | 328 |
| Tertile 3 | 145 | 863 | 559 | 401 | 3968 | 497 | 672 | 943 |
| Total | 436 | 410 | 462 | 11 | 3968 | 126 | 277 | 495 |
|  |  |  |  |  |  |  |  |  |
| CIT |  |  |  |  |  |  |  |  |
| Tertile 1 | 146 | 14 | 4 | 4 | 21 | 10 | 14 | 18 |
| Tertile 2 | 145 | 38 | 14 | 22 | 75 | 26 | 34 | 44 |
| Tertile 3 | 145 | 344 | 385 | 75 | 2885 | 124 | 203 | 410 |
| Total | 436 | 132 | 268 | 4 | 2885 | 18 | 34 | 124 |
|  |  |  |  |  |  |  |  |  |
| DON |  |  |  |  |  |  |  |  |
| Tertile 1 | 146 | 103 | 23 | 48 | 141 | 87 | 102 | 121 |
| Tertile 2 | 145 | 176 | 22 | 141 | 214 | 156 | 174 | 196 |
| Tertile 3 | 145 | 414 | 452 | 215 | 4967 | 261 | 311 | 411 |
| Total | 436 | 231 | 293 | 48 | 4967 | 121 | 173 | 261 |

Abbreviations: OTA, Ochratoxin A; CIT, Citrinin; DON, Deoxynivalenol; Min, Minimum; Max, Maximum; p25, 25^th^ percentile; p50, 50^th^ percentile; p75, 75^th^ percentile

**Supplementary Table S3: Association between adverse birth outcomes and higher maternal dietary intake of ochratoxin A, among pregnant women in rural Habiganj district, Bangladesh: Breaking the highest tertile into finer groups**

| **Mycotoxin intake** |  | | **Low birth weight (N=317)** |
| --- | --- | --- | --- |
| **Category of Ochratoxin A intake (ng/kg bw)** | **n** | | **Adjusted*^b^* OR (95% CI)** |
| (11 – 174) | 96 | | Reference |
| (175 – 400) | 112 | | 1.06 (0.39, 2.89) |
| (401 – 1000) | 83 | | 2.03 (0.71, 5.82) |
| (1001 – 1500) | 12 | | 4.05 (0.62, 26.7) |
| (1501 – 3968) | 14 | | 6.59 (1.26, 34.5) |
| *P* for trend |  |  | **0.009** |

^b^Adjusted for mothers' age at enrolment, household wealth index, mother's educational attainment, low maternal weight (mother’s weight at enrolment<55kg), average dietary diversity score, household food security status, parity, history of previous pregnancy loss, the season of sample collection, antenatal attendance, iron supplementation, infant sex, length at birth, and gestational age at birth detection of more than one mycotoxin, and clustering at the settlement and woman levels

**Supplementary Table S4: Distribution of maternal single or co-exposures to specific mycotoxins and adverse birth outcomes in a cohort of pregnant women in rural Habiganj district, Bangladesh**

| **Specific mycotoxins detected above LOD** | **Pregnancy loss**  **(N=436)** | | |  | **Preterm birth**  **(N=317)** | | |  | **Low birth weight (N=317)** | | |  | **Small for gestational age (N=317)** | | |  | **Small-vulnerable newborn (N=317)** | | |
| --- | --- | --- | --- | --- | --- | --- | --- | --- | --- | --- | --- | --- | --- | --- | --- | --- | --- | --- | --- |
|  | No  n (%) | Yes  n (%) | Total (%) |  | No  n (%) | Yes  n (%) | Total (%) |  | No  n (%) | Yes  n (%) | Total (%) |  | No  n (%) | Yes  n (%) | Total (%) |  | No  n (%) | Yes  n (%) | Total (%) |
|  |  |  |  |  |  |  |  |  |  |  |  |  |  |  |  |  |  |  |  |
| None | 13 | 3 | 16 |  | 8 | 1 | 9 |  | 7 | 2 | 9 |  | 6 | 3 | 9 |  | 6 | 3 | 9 |
|  | (81.3) | (18.7) | (100) |  | (88.9) | (11.1) | (100) |  | (77.8) | (22.2) | (100) |  | (66.7) | (33.3) | (100) |  | (66.7) | (33.3) | (100) |
|  |  |  |  |  |  |  |  |  |  |  |  |  |  |  |  |  |  |  |  |
| OTA alone | 128 | 12 | 140 |  | 85 | 15 | 100 |  | 80 | 20 | 100 |  | 62 | 38 | 100 |  | 50 | 50 | 100 |
|  | (91.4) | (8.6) | (100) |  | (85) | (15) | (100) |  | (80) | (20) | (100) |  | (62) | (38) | (100) |  | (50) | (50) | (100) |
|  |  |  |  |  |  |  |  |  |  |  |  |  |  |  |  |  |  |  |  |
| CIT alone | 6 | 0 | 6 |  | 3 | 1 | 4 |  | 1 | 3 | 4 |  | 2 | 2 | 4 |  | 1 | 3 | 4 |
|  | (100) | (0) | (100) |  | (75) | (25) | (100) |  | (25) | (75) | (100) |  | (50) | (50) | (100) |  | (25) | (75) | (100) |
|  |  |  |  |  |  |  |  |  |  |  |  |  |  |  |  |  |  |  |  |
| OTA+CIT | 218 | 19 | 237 |  | 142 | 35 | 177 |  | 123 | 54 | 177 |  | 115 | 62 | 177 |  | 84 | 93 | 177 |
|  | (92) | (8) | (100) |  | (80.2) | (19.8) | (100) |  | (69.5) | (30.5) | (100) |  | (65) | (35) | (100) |  | (47.5) | (52.5) | (100) |
|  |  |  |  |  |  |  |  |  |  |  |  |  |  |  |  |  |  |  |  |
| OTA+DON/AF/FB1 | 11 | 2 | 13 |  | 10 | 0 | 10 |  | 10 | 0 | 10 |  | 6 | 4 | 10 |  | 6 | 4 | 10 |
|  | (84.6) | (15.4) | (100) |  | (100) | (0) | (100) |  | (100) | (0) | (100) |  | (60) | (40) | (100) |  | (60) | (40) | (100) |
|  |  |  |  |  |  |  |  |  |  |  |  |  |  |  |  |  |  |  |  |
| OTA+CIT+DON | 16 | 2 | 18 |  | 12 | 2 | 14 |  | 13 | 1 | 14 |  | 12 | 2 | 14 |  | 10 | 4 | 14 |
|  | (88.9) | (11.1) | (100) |  | (85.7) | (14.3) | (100) |  | (92.9) | (7.1) | (100) |  | (85.7) | (14.3) | (100) |  | (71.4) | (28.6) | (100) |
|  |  |  |  |  |  |  |  |  |  |  |  |  |  |  |  |  |  |  |  |
| OTA+CIT+AF/ZEN | 4 | 2 | 6 |  | 3 | 0 | 3 |  | 2 | 1 | 3 |  | 1 | 2 | 3 |  | 1 | 2 | 3 |
|  | (66.7) | (33.3) | (100) |  | (100) | (0) | (100) |  | (66.7) | (33.3) | (100) |  | (33.3) | (66.7) | (100) |  | (33.3) | (66.7) | (100) |
|  |  |  |  |  |  |  |  |  |  |  |  |  |  |  |  |  |  |  |  |

Abbreviations: LOD, Limit of Detection; FB_1_, Fumonisin B_1_; OTA, Ochratoxin A; CIT, Citrinin; DON, Deoxynivalenol; AF, Aflatoxin; ZEN, zearalenone
